# Supplementary material for: Multidimensional tie strength and economic development
Source: Sci Rep. 2022 Dec 21;12:22081. doi: 10.1038/s41598-022-26245-4 (PMC9772415; doi:10.1038/s41598-022-26245-4)
Supplement: Supplementary file 1 — Supplementary Information. [file 41598_2022_26245_MOESM1_ESM.pdf]

Supplementary Information to “Multidimensional Tie Strength and Economic Development”

Network distributions

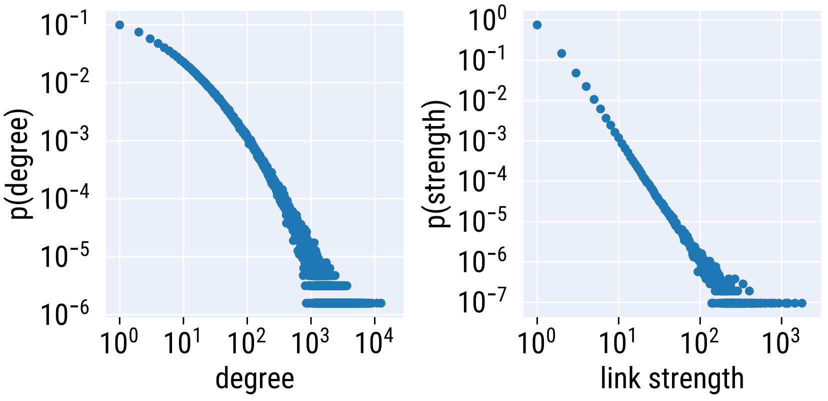

Figure SI1. Distributions of node degree (left) and link strength calculated as the number of messages flowing between connected nodes (right) in the communication graph $\mathscr{G}$.

The distributions of the degree and strength of the Reddit communication network are shown in Figure SI1.

Regressions without population density

Predicting GDP per capita from:

Diversity on full communication graph				Spatial diversity on dimension-specific graphs			
**Feature**	β	**SE**	p	**Feature**	β	**SE**	p
α (intercept)	0.182	0.093	0.058	α (intercept)	0.244	0.070	0.001
$D_{spatial}$	0.436	0.154	0.007	$D^{knowledge}_{spatial}$	1.168	0.188	0.000
				$D^{support}_{spatial}$	-0.593	0.180	0.002
Durbin-Watson stat. = 2.280	R^2_{adj} = **0.14**			Durbin-Watson stat. = 1.983	R^2_{adj} = **0.48**		

Table SI1. Linear regressions to predict GDP per capita of US states from: (left) spatial diversity computed on the full communication graph; (right) spatial diversities computed on dimension-specific communication graphs. Adjusted R^2 and Durbin-Watson statistic for autocorrelation (values close to 2 indicate no autocorrelation) are reported. The contribution of individual features to the models is described by their *beta*-coefficients, standard errors (SE) and p-values.

Table SI1 shows the results of regression models that include spatial diversity but exclude population density as a control variable. For reference, a model that predicts GDP from population density only achieved an R^2_{adj} of 0.26.

Spatial vs. social diversity

Table SI2 reports the results of linear regressions that include social diversity, alone or combined with spatial diversity. The combination of $D^{knowledge}_{social}$ and $D^{support}_{spatial}$ yields the best fit ($R^2_{adj} = 0.64$), only slightly above the model that considers only spatial diversity ($R^2_{adj} = 0.62$ Table 1).

Sensitivity to minimum edge weight and classifier threshold

We included in the conversation graph $\mathscr{G}$ only edges with minimum weight of 4. This was the optimal threshold we found in the range $[1,6]$ (Figure SI2, left). Similarly, the dimension-specific graphs $\mathscr{G}_d$ are obtained after setting a threshold θ_d equal to the value of the α percentile on the distribution of the classifier scores s_d. We explored different values of α and found that the 99th percentile works best (Figure SI2, right). However, dimension-specific graphs created using any percentiles above the 75th yielded better prediction than any version of the full conversation graph.

Predicting GDP per capita from:

Social+Spatial diversity on full graph				Social diversity on dimension-specific graphs			
**Feature**	β	**SE**	p	**Feature**	β	**SE**	p
α (intercept)	0.065	0.094	0.493	α (intercept)	0.193	0.066	0.006
Pop. density	0.581	0.159	0.001	Pop. density	0.440	0.121	0.001
D_{social}	0.489	0.190	0.014	$D_{social}^{knowledge}$	0.884	0.147	0.000
$D_{spatial}$	-0.031	0.179	0.864	$D_{social}^{support}$	-0.435	0.142	0.004
Durbin-Watson stat. = 2.108	R^2_{adj} = **0.38**			Durbin-Watson stat. = 2.131	R^2_{adj} = **0.60**		

Social+spatial on dimension-specific graphs				Social+spatial on dimension-specific graphs			
**Feature**	β	**SE**	p	**Feature**	β	**SE**	p
α (intercept)	0.190	0.059	0.003	α (intercept)	0.183	0.069	0.012
Pop. density	0.492	0.113	0.000	Pop. density	0.446	0.127	0.001
$D_{social}^{knowledge}$	1.052	0.159	0.000	$D_{spatial}^{knowledge}$	0.8081	0.148	0.000
$D_{spatial}^{support}$	-0.585	0.152	0.000	$D_{social}^{support}$	-0.339	0.141	0.021
Durbin-Watson stat. = 2.110	R^2_{adj} = **0.64**			Durbin-Watson stat. = 2.027	R^2_{adj} = **0.56**		

Table SI2. Linear regressions to predict GDP per capita of US states from social diversity in the full graph (top, left), social diversity computed on dimension-specific communication graphs (top, right), and combinations of social and spatial diversity on the on dimension-specific communication graphs (bottom), left and right. Population density is added as a control variable. Adjusted R^2 and Durbin-Watson statistic for autocorrelation (values close to 2 indicate no autocorrelation) are reported. The contribution of individual features to the models is described by their *beta*-coefficients, standard errors (SE) and p-values.

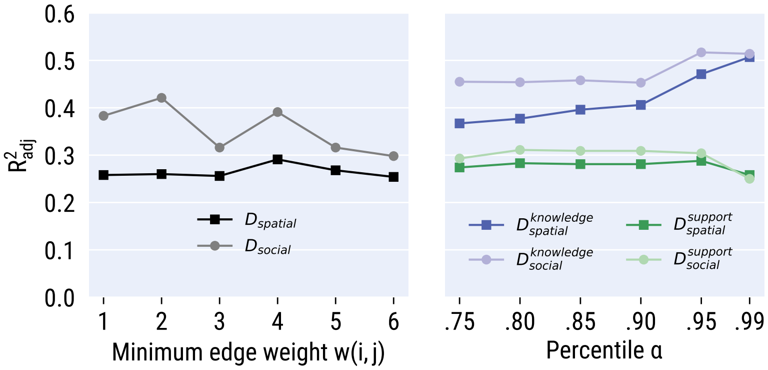

Figure SI2. Left: adjusted R^2 of linear regressions that predict GDP from diversity on the full graph ($D_{spatial}$, D_{social}) as the minimum weight w of edges included in the graph varies. Right: adjusted R^2 of univariate linear regressions to predict GDP from social and spatial diversity on the *knowledge* graph and *support* graphs as the percentile α used to binarize the classifier scores varies. We included population density as control variable in all regressions.

Sensitivity to set of states

We restricted our study to a subset of 44 states, after removing states whose Reddit penetration was too low or was not proportional to the population of residents. The inclusion of outlier states is detrimental to the performance of all models, yet the quality of fit of the *knowledge*-specific model is far superior to the one obtained using the full graph—two to three times as good. Multivariate regressions with all 50 states are presented in Table SI3; their predictive power is roughly halved compared to that of the models fit on 44 states only. Yet, the dimension-specific model was still 130% more accurate than the one using diversity computed on the full conversation graph.

Predicting GDP per capita from:

**Population density**			
**Feature**	β	**SE**	p
α (intercept)	0.393	0.042	0.000
Pop. density	0.419	0.154	0.009
Durbin-Watson stat. = 2.150	R^2_{adj} = **0.12**		

**Diversity on full communication graph**			
**Feature**	β	**SE**	p
α (intercept)	0.264	0.093	0.007
Pop. density	0.372	0.164	0.028
D_{social}	0.271	0.181	0.142
$D_{spatial}$	-0.014	0.174	0.934
Durbin-Watson stat. = 2.175	R^2_{adj} = **0.13**		

**Diversity on dimension-specific graphs**			
**Feature**	β	**SE**	p
α (intercept)	0.499	0.101	0.000
Pop. density	0.303	0.142	0.039
$D_{social}^{knowledge}$	0.661	0.171	0.000
$D_{spatial}^{support}$	-0.555	0.202	0.009
Durbin-Watson stat. = 2.339	R^2_{adj} = **0.30**		

Table SI3. Linear regressions to predict GDP per capita of 50 US states from: (left) population density only; (center) spatial and social diversity computed on the full communication graph; (right) spatial and social diversity computed on dimension-specific communication graphs. The variables $D_{social}^{knowledge}$ and $D_{spatial}^{support}$ were picked automatically by a feature-selection algorithm out of all the dimension-specific diversity measures. Population density is added as a control variable in the latter two models. Adjusted R^2 and Durbin-Watson statistic for autocorrelation (values close to 2 indicate no autocorrelation) are reported. The contribution of individual features to the models is described by their *beta*-coefficients, standard errors (SE) and p-values.

Baseline with random selection of links

We calculated univariate regressions based on a randomized model. Specifically, we predicted GDP from diversity measures calculated on a communication graph created from a random selection of 1% of the messages. This is equivalent of creating a null model where the *knowledge* or *support* labels of messages are reshuffled at random, such that the association between social links and social dimensions is disrupted. We repeated the experiment for 50 random runs. For $D_{spatial}^{random}$, we obtained $\bar{R}^2_{adj} = 0.084$, $(stdev = 0.090)$; for $D_{spatial}^{random}$, we obtained $\bar{R}^2_{adj} = 0.096$, $(stdev = 0.101)$. The R^2 of these random models are much lower than those obtained considering 1% of *knowledge* or *support* messages ($R^2_{adj} = 0.35$ and $R^2_{adj} = 0.26$, respectively).

Sensitivity to temporal window

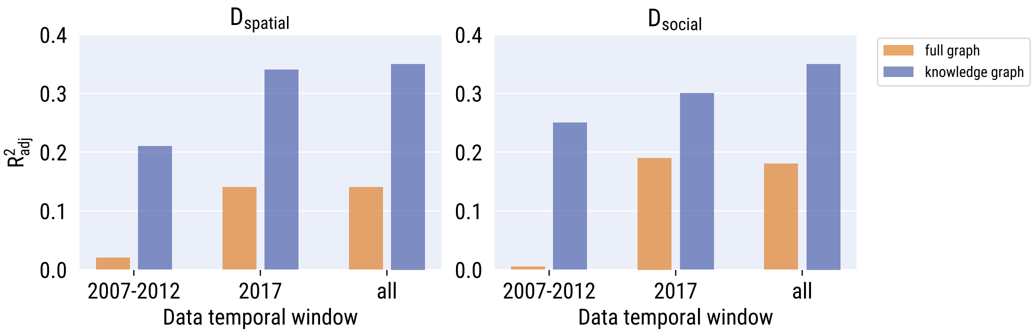

Figure SI3. Sensitivity of data temporal window. Adjusted R^2 of univariate linear regressions to predict GDP from social and spatial diversity from three temporal windows: 2007 to the end of 2012, 2017, and the full dataset. Results for the full conversation graph and for the knowlegde graph are compared.

The Reddit data we collected comes from two distinct time periods: from 2007 to the end of 2012 and during the whole year of 2017. In our study, we built conversation graphs with the data from these two periods jointly (all). Later, we explored how conversation graphs made from each period individually are predictive of GDP in year 2017. Figure SI3 shows the adjusted R^2 of univariate linear regressions to predict GDP from social and spatial diversity, using either the full conversation graph or the *knowledge* graph. The most recent data from year 2017 best approximated the results obtained using all the data. The performance decay in the earliest temporal window of years 2007-2012 was conspicuous for models based on the full graph: their R^2 dropped close to zero. The performance decay should not be attributed to data sparsity because, in our dataset, posts published in the period 2007-2012 are more abundant than those published in 2017 (9.8M vs. 6.4M).

This result suggests that *knowledge*-specific interactions not only better predict economic development; they also provide

a predictive signal that is more resilient to temporal shifts of the data relative to the time in which the outcome variable was measured.

Sensitivity to threshold for user geo-referencing

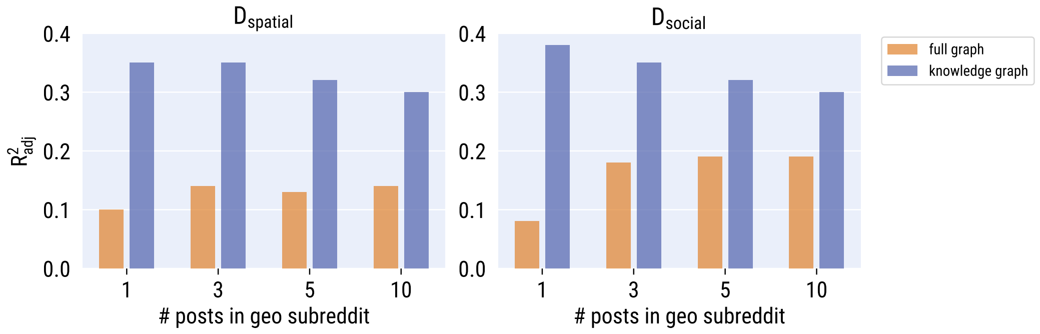

Figure SI4. Adjusted R^2 of regression models whose variables are obtained from conversation graphs including different sets of geo-referenced users. Specifically, we included only users who posted a minimum number of comments or submissions in geo-salient subreddits. Results for the full conversation graph and for the knowlegde graph are compared.

To geo-reference Reddit users, we analyzed their activity in geo-salient subreddits. Specifically, we assigned a user to a US state if they posted at least 3 submissions or comments in subreddits related to that state. We explored how the prediction results change by varying this threshold. Figure SI4 shows the adjusted R^2 of univariate linear regressions to predict GDP from social and spatial diversity, using either the full conversation graph or the *knowledge* graph. Results are broken down by different thresholds of minimum number of geo-salient posts required for assigning a user to a geographical location. Raising the threshold is beneficial to the model based on the full communication graph, as it helps filtering out incorrect user-state associations. The R^2 on the *knowledge* network slightly declines as the threshold increases; this is likely due to the fact that further filtering on a network that contains only 1% of links aggravates data sparsity.

Distributions of diversity scores

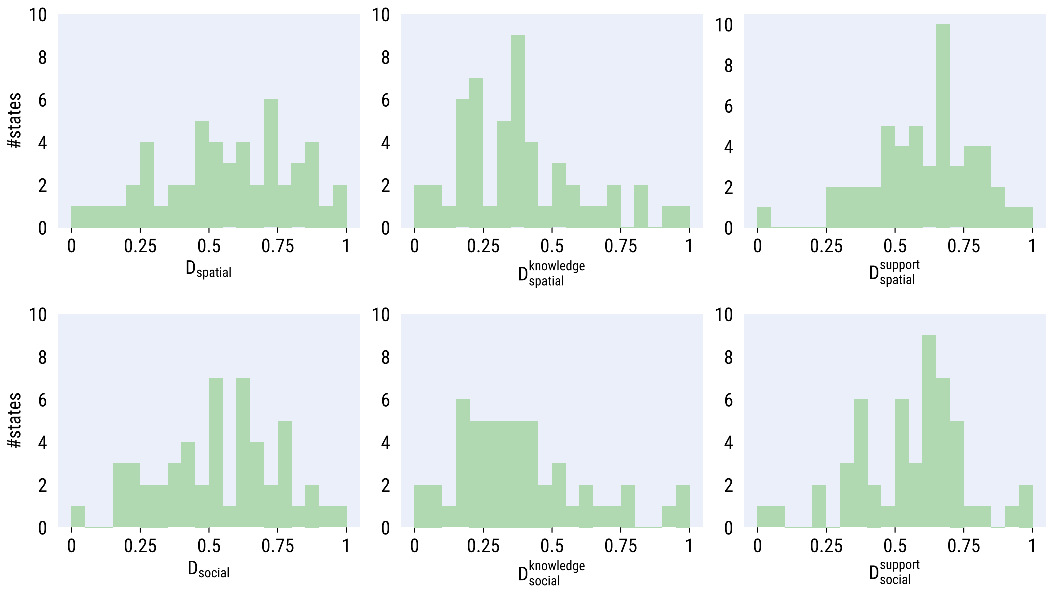

Figure SI5. Distribution of diversity scores calculated on the full graph ($D_{spatial}$, D_{social}) and on the *knowledge* and *support* graphs ($D_{spatial}^{knowledge}$, $D_{social}^{knowledge}$, $D_{spatial}^{support}$, $D_{social}^{support}$). Values are min-max normalized.

In Figure SI5, we report the distribution of diversity scores across US states calculated on the full graph and on the *knowledge* graph.

Multivariate models with alternative sets of variables

Predicting GDP per capita				Predicting GDP per capita			
**Feature**	β	**SE**	p	**Feature**	β	**SE**	p
α (intercept)	0.219	0.130	0.102	α (intercept)	0.172	0.140	0.229
Pop. density	0.471	0.164	0.007	Pop. density	0.487	0.157	0.004
$D_{social}^{knowledge}$	0.748	0.510	0.153	$D_{spatial}^{knowledge}$	0.774	0.578	0.190
$D_{social}^{support}$	-0.445	0.279	0.120	$D_{spatial}^{support}$	-0.671	0.316	0.042
$D_{social}^{conflict}$	-0.117	0.300	0.700	$D_{spatial}^{conflict}$	-0.136	0.336	0.688
D_{social}^{status}	0.047	0.266	0.860	$D_{spatial}^{status}$	0.183	0.359	0.615
D_{social}^{power}	0.169	0.366	0.648	$D_{spatial}^{power}$	0.050	0.391	0.900
D_{social}^{trust}	-0.074	0.264	0.780	$D_{spatial}^{trust}$	-0.096	0.302	0.752
$D_{social}^{similarity}$	-0.049	0.365	0.895	$D_{spatial}^{similarity}$	0.071	0.445	0.874
$D_{social}^{identity}$	0.214	0.329	0.520	$D_{spatial}^{identity}$	0.294	0.318	0.363
D_{social}^{fun}	0.117	0.300	0.698	$D_{spatial}^{fun}$	0.117	0.346	0.737
$D_{social}^{romance}$	-0.233	0.278	0.409	$D_{spatial}^{romance}$	-0.076	0.337	0.822
Durbin-Watson stat. = 1.998		R_{adj}^2 = **0.53**		Durbin-Watson stat. = 1.985		R_{adj}^2 = **0.55**	

Table SI4. Linear regressions to predict GDP per capita of US states from the social and spatial diversity (D_{social}^d, $D_{spatial}^d$) computed on dimension-specific communication graphs. Population density is added as a control variable. Adjusted R^2 and Durbin-Watson statistic for autocorrelation (values close to 2 indicate no autocorrelation) are reported. The contribution of individual features to the models is described by their *beta*-coefficients, standard errors (SE) and *p*-values.

Knowledge and *support* might not be the only two social dimensions associated to economic development. To systematically evaluate how this association varies when considering a wider set of dimensions, we ran two linear regressions that include the social and spatial diversities of all the social dimensions that our NLP tool can capture, plus population density as control (Table SI4). The adjusted R^2 of these models reach 0.55, which is lower than models considering *knowledge* and *support* only.

These models include too many features, considering the limited number of datapoints (44 states). As a result, the coefficients of all variables are not statistically significant ($p > 0.1$). When the set of independent variables is large, it is common practice to use feature-selection approaches to select only those variables that explain most of the variability of the outcome variable. In our experiments we used *stepAIC*. This method is based on the Akaike Information Criterion, or AIC for short (see Sakamoto et al., *"Akaike information criterion statistics"*, 1986), an estimate of the relative amount of information lost by a model to represent the process that generated the empirical data. The AIC score rewards models that achieve a high goodness-of-fit score and penalizes them if they become overly complex. stepAIC measures the AIC score of models obtained by removing different sets of features from the original model and selects the feature combination that yields the lowest AIC. Automatic feature selection kept two variables: social diversity of *knowledge*, and spatial diversity of *support*. This reduced model (summarized in Table SI2) yielded an R_{adj}^2 of 0.64, which is only slightly better than the model that considers only the spatial diversity of *knowledge* and *support* (Table 1).
